# Supplementary material for: The Systems Biology Research Tool: evolvable open-source software
Source: BMC Syst Biol. 2008 Jun 29;2:55. doi: 10.1186/1752-0509-2-55 (PMC2446383; doi:10.1186/1752-0509-2-55)
Supplement: Additional file 1 — SBRT Archive. An archive of the current version of the Systems Biology Research Tool. [file 1752-0509-2-55-S1.zip › sbrt-1.4.0/doc/developers_guide/api/sbrt/kernel/fba/IrrevRxnFluxome.html]

IrrevRxnFluxome


|  |  |  |
| --- | --- | --- |
| **Overview** \| **Package** \| **Class** \| **Use** \| **Tree** \| **Deprecated** \| **Index** \| **Help** | |  |
| **PREV CLASS**   **NEXT CLASS** | **FRAMES**    **NO FRAMES**     **All Classes** |  |
| SUMMARY: NESTED \| FIELD \| CONSTR \| METHOD | DETAIL: FIELD \| CONSTR \| METHOD |  |


---


## sbrt.kernel.fba Interface IrrevRxnFluxome

**All Superinterfaces:**: CatalyzedFluxome, Fluxome

**All Known Implementing Classes:**: IrrevRxnFluxomeV1

---

``` public interface IrrevRxnFluxome extends CatalyzedFluxome ```

This class is used to represent fluxomes that contain only irreversible
reactions. All internal reactions must be of the type `IrrevRxn`, and
all exchange reactions must be of the types `SourceRxn` and
`SinkRxn`. This sort of representation offers a variety of advantages
over more commonly used models. It allows one to constrain all forward and
reverse reactions independently of each other, and it also means that
the flux space formed by this fluxome lies entirely in the positive orthant.

For example, an organism like yeast can consume ethanol as a food source or
produce it as a waste product, depending on a variety of factors. Breaking
apart the ethanol-associated exchange reactions into forward and reverse
(that is, source and sink) reactions allows one to model this phenomenon in
much finer detail.

**Author:**
:   This class was written by Jeremiah Wright while in the Wagner lab.

---

| **Method Summary** | |
| --- | --- |
| `void` | `checkRxnName(java.lang.String reactionName)`             Ensures the provided string is a valid reaction name. |
| `void` | `checkRxnNames(java.util.Collection<java.lang.String> rxnNames)`             Ensures each element of the provided collection is a valid reaction name. |
| `Or<And<java.lang.String>>` | `getCatalystRule(java.lang.String rxnName)`             Returns the boolean logic statement for the catalysts of the reaction with the provided name. |
| `java.util.Map<java.lang.String,Or<And<java.lang.String>>>` | `getCatalystRules()`             Returns all of the boolean logic statements regarding reaction catalysis. |
| `java.util.Map<java.lang.String,IrrevRxn>` | `getEquivRxns(java.lang.String reactionName)`             Returns the set reactions in this fluxome that are stoichiometrically equivalent to the provided reaction. |
| `IrrevRxn` | `getInternalRxn(java.lang.String rxnName)`             Returns the internal reaction in this fluxome with the provided name. |
| `java.util.Map<java.lang.String,IrrevRxn>` | `getInternalRxns()`             Returns the set of all internal reactions in this fluxome. |
| `java.util.Map<java.lang.String,IrrevRxn>` | `getInternalRxns(java.util.Collection<java.lang.String> rxnNames)`             Returns the internal reactions with the provided names. |
| `java.util.Map<java.lang.String,ChemRxn>` | `getReverseRxns(java.lang.String reactionName)`             Returns the set reactions in this fluxome that are stoichiometrically equivalent to the provided reaction in reverse. |
| `SinkRxn` | `getSinkRxn(java.lang.String rxnName)`             Returns the sink reaction in this fluxome with the provided name. |
| `java.util.Set<java.lang.String>` | `getSinkRxnNames()`             Returns the set of names of all sink reactions in this fluxome. |
| `java.util.Map<java.lang.String,SinkRxn>` | `getSinkRxns()`             Returns the set of all sink reactions in this fluxome. |
| `java.util.Map<java.lang.String,SinkRxn>` | `getSinkRxns(java.util.Collection<java.lang.String> rxnNames)`             Returns the sink reactions with the provided names. |
| `SourceRxn` | `getSourceRxn(java.lang.String rxnName)`             Returns the source reaction in this fluxome with the provided name. |
| `java.util.Set<java.lang.String>` | `getSourceRxnNames()`             Returns the set of names of all source reactions in this fluxome. |
| `java.util.Map<java.lang.String,SourceRxn>` | `getSourceRxns()`             Returns the set of all source reactions in this fluxome. |
| `java.util.Map<java.lang.String,SourceRxn>` | `getSourceRxns(java.util.Collection<java.lang.String> rxnNames)`             Returns the source reactions with the provided names. |
| `IrrevRxnFluxome` | `replaceConstraints(FluxConstraints constraints, double constraintTolerance)`             Returns a copy of this fluxome, but with all constraints replaced by those provided. |
| `int` | `sinkRxns()`             Returns the total number of sink reactions contained in this fluxome. |
| `int` | `sourceRxns()`             Returns the total number of source reactions contained in this fluxome. |

**Methods inherited from interface sbrt.kernel.fba.CatalyzedFluxome**

`areReqCatalysts, catalysts, getCatalysts, getRxnNames, getRxns, isCatalyst, isReqCatalyst`

**Methods inherited from interface sbrt.kernel.fba.Fluxome**

`addedConstraints, areRxnNames, exchangeRxns, getConstraints, getEquivRxnNames, getEquivRxnNames, getExchangeRxnNames, getExchangeRxns, getFluxConstraint, getInternalRxnNames, getReverseRxnNames, getRxn, getRxnExprConstraint, getRxnNames, getRxns, getRxns, getSpecies, getStoichiometryMatrix, internalRxns, isFluxVector, isRxnName, isSpecies, isValid, redundantRxns, rxns, species`

**Method Detail**

### checkRxnNames

```
void checkRxnNames(java.util.Collection<java.lang.String> rxnNames)
```

:   Ensures each element of the provided collection is a valid reaction name.

    :   **Parameters:**: `rxnNames` - a collection of strings. **Throws:**: `java.lang.IllegalArgumentException` - if any element of this collection is not a valid reaction name.: `java.lang.NullPointerException` - if the collection is null.

---


### checkRxnName

```
void checkRxnName(java.lang.String reactionName)
```

:   Ensures the provided string is a valid reaction name.

    :   **Parameters:**: `reactionName` - a potential reaction name. **Throws:**: `java.lang.IllegalArgumentException` - if the provided string is not a valid reaction name.

---


### getInternalRxns

```
java.util.Map<java.lang.String,IrrevRxn> getInternalRxns()
```

:   Returns the set of all internal reactions in this fluxome.

    :   **Specified by:**: `getInternalRxns` in interface `Fluxome`
    :   **Returns:**: the set of all internal reactions in this fluxome.

---


### getInternalRxns

```
java.util.Map<java.lang.String,IrrevRxn> getInternalRxns(java.util.Collection<java.lang.String> rxnNames)
```

:   Returns the internal reactions with the provided names.

    :   **Parameters:**: `rxnNames` - the names of the reactions to be returned. **Returns:**: reaction names mapped to their reaction.

---


### getReverseRxns

```
java.util.Map<java.lang.String,ChemRxn> getReverseRxns(java.lang.String reactionName)
```

:   Returns the set reactions in this fluxome that are
    stoichiometrically equivalent to the provided reaction in reverse.

    :   **Parameters:**: `reactionName` - the name of the reaction for which the stoichiometrically equivalent reverse reactions will be returned. **Returns:**: the set of reactions in this fluxome that are stoichiometrically equivalent to the provided reaction in reverse.

---


### getEquivRxns

```
java.util.Map<java.lang.String,IrrevRxn> getEquivRxns(java.lang.String reactionName)
```

:   Returns the set reactions in this fluxome that are
    stoichiometrically equivalent to the provided reaction.

    :   **Parameters:**: `reactionName` - the name of the reaction for which the stoichiometrically equivalent reactions will be returned. **Returns:**: the set of reactions in this fluxome that are stoichiometrically equivalent to the provided reaction.

---


### getSourceRxnNames

```
java.util.Set<java.lang.String> getSourceRxnNames()
```

:   Returns the set of names of all source reactions in this fluxome.

    :   **Returns:**: the set of names of all source reactions in this fluxome.

---


### getSourceRxns

```
java.util.Map<java.lang.String,SourceRxn> getSourceRxns(java.util.Collection<java.lang.String> rxnNames)
```

:   Returns the source reactions with the provided names.

    :   **Parameters:**: `rxnNames` - the names of the reactions to be returned. **Returns:**: reaction names mapped to their reaction.

---


### getSinkRxnNames

```
java.util.Set<java.lang.String> getSinkRxnNames()
```

:   Returns the set of names of all sink reactions in this fluxome.

    :   **Returns:**: the set of names of all sink reactions in this fluxome.

---


### getSinkRxns

```
java.util.Map<java.lang.String,SinkRxn> getSinkRxns(java.util.Collection<java.lang.String> rxnNames)
```

:   Returns the sink reactions with the provided names.

    :   **Parameters:**: `rxnNames` - the names of the reactions to be returned. **Returns:**: reaction names mapped to their reaction.

---


### getSourceRxns

```
java.util.Map<java.lang.String,SourceRxn> getSourceRxns()
```

:   Returns the set of all source reactions in this fluxome.

    :   **Returns:**: the names of all source reactions mapped to their corresponding reaction.

---


### getSinkRxns

```
java.util.Map<java.lang.String,SinkRxn> getSinkRxns()
```

:   Returns the set of all sink reactions in this fluxome.

    :   **Returns:**: the names of all sink reactions mapped to their corresponding reaction.

---


### getInternalRxn

```
IrrevRxn getInternalRxn(java.lang.String rxnName)
```

:   Returns the internal reaction in this fluxome with the provided name.

    :   **Parameters:**: `rxnName` - the name of the internal reaction to return. **Returns:**: the internal reaction in this fluxome with the provided name.

---


### getSourceRxn

```
SourceRxn getSourceRxn(java.lang.String rxnName)
```

:   Returns the source reaction in this fluxome with the provided name.

    :   **Parameters:**: `rxnName` - the name of the source reaction to return. **Returns:**: the source reaction in this fluxome with the provided name.

---


### getSinkRxn

```
SinkRxn getSinkRxn(java.lang.String rxnName)
```

:   Returns the sink reaction in this fluxome with the provided name.

    :   **Parameters:**: `rxnName` - the name of the sink reaction to return. **Returns:**: the sink reaction in this fluxome with the provided name.

---


### sourceRxns

```
int sourceRxns()
```

:   Returns the total number of source reactions contained
    in this fluxome.

    :   **Returns:**: the total number of source reactions contained in this fluxome.

---


### sinkRxns

```
int sinkRxns()
```

:   Returns the total number of sink reactions contained
    in this fluxome.

    :   **Returns:**: the total number of sink reactions contained in this fluxome.

---


### replaceConstraints

```
IrrevRxnFluxome replaceConstraints(FluxConstraints constraints,
                                   double constraintTolerance)
```

:   Returns a copy of this fluxome, but with all constraints
    replaced by those provided. If a flux constraint for a
    particular reaction was not provided, its default value
    will be used.

    :   **Specified by:**: `replaceConstraints` in interface `CatalyzedFluxome` **Specified by:**: `replaceConstraints` in interface `Fluxome`
    :   **Parameters:**: `constraints` - the new set of flux constraints on the system.: `constraintTolerance` - the allowed amount by which constraints can violate their maximum defined intervals. These maximum defined intervals are implementation-dependent. **Returns:**: a copy of this fluxome, but with all constraints replaced by those provided.

---


### getCatalystRule

```
Or<And<java.lang.String>> getCatalystRule(java.lang.String rxnName)
```

:   Returns the boolean logic statement for the catalysts of the reaction
    with the provided name.

    :   **Specified by:**: `getCatalystRule` in interface `CatalyzedFluxome`
    :   **Parameters:**: `rxnName` - the name of the reaction for which the catalyst rules are to be returned. **Returns:**: the boolean logic statement for the catalysts of the reaction with the provided name. If the specified reaction is not catalyzed, an empty (non-null) rule is returned.

---


### getCatalystRules

```
java.util.Map<java.lang.String,Or<And<java.lang.String>>> getCatalystRules()
```

:   Returns all of the boolean logic statements regarding reaction catalysis.

    :   **Specified by:**: `getCatalystRules` in interface `CatalyzedFluxome`
    :   **Returns:**: the names of catalyzed reactions mapped to their boolean logic rules of their catalyst(s).


---


|  |  |  |
| --- | --- | --- |
| **Overview** \| **Package** \| **Class** \| **Use** \| **Tree** \| **Deprecated** \| **Index** \| **Help** | |  |
| **PREV CLASS**   **NEXT CLASS** | **FRAMES**    **NO FRAMES**     **All Classes** |  |
| SUMMARY: NESTED \| FIELD \| CONSTR \| METHOD | DETAIL: FIELD \| CONSTR \| METHOD |  |


---
